# Crystal structure of 4-(prop-2-yn-1-yl­oxy)benzo­nitrile

**DOI:** 10.1107/S2056989014028035

**Published:** 2015-01-10

**Authors:** Mayu Kanagawa, Tsunehisa Okuno

**Affiliations:** a Department of Material Science and Chemistry, Wakayama University, Sakaedani, Wakayama, 640-8510, Japan

**Keywords:** crystal structure, prop-2-yn-1-yl­oxy, hydrogen bonding, C—H⋯π inter­actions, π–π stacking inter­actions

## Abstract

In the title compound, C_10_H_7_NO, the dihedral angle between the aromatic ring and the prop-2-yn-1-yl­oxy grouping is 9.47 (10)°. The bond lengths indicate electronic conjugation between the cyano group, the benzene ring and the propyn­yloxy oxygen atom. In the crystal, a hydrogen bond between the acetyl­enic C—H atom and the cyano nitro­gen atom link the mol­ecules into wave-like [30-1] C(11) chains. These chains are connected by C*sp* ^2^—H⋯π_ac_ (π_ac_ is the acetyl­inic C—C triple bond) close contacts [2.794 (1) Å], resulting in a rolling sheet structure parallel to the *ac* plane and aromatic π–π stacking inter­actions between the sheets [centroid–centroid distance = 3.593 (2) Å] generate a three-dimensional network.

## Related literature   

The title compound is an aryl propargyl ether derivative which attracts inter­est with regard to Claisen rearrangement (Kenny *et al.* 2006 ▸; Wang *et al.* 2012 ▸) or cleavage of the O–CH_2_ bond by boron reagents (Yao *et al.* 2009 ▸). For related structures of 4-(prop-2-yn-1-yl­oxy)benzenes, see: Lindeman *et al.* (1993 ▸); Zhu *et al.* (2006 ▸); Zhang *et al.* (2008 ▸); Marsh (2009 ▸); Ranjith *et al.* (2010 ▸); Li *et al.* (2009 ▸); Ao *et al.* (2011 ▸); Al-Mehana *et al.* (2011 ▸); Belay *et al.* (2012 ▸); Doi & Okuno (2013 ▸).
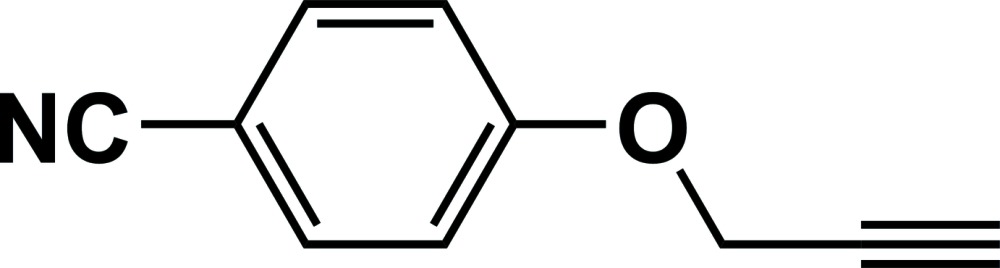


## Experimental   

### Crystal data  

C_10_H_7_NO
*M* *_r_* = 157.17
Monoclinic, 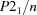

*a* = 6.033 (4) Å
*b* = 7.393 (5) Å
*c* = 17.527 (11) Å
β = 90.836 (11)°
*V* = 781.7 (9) Å^3^
*Z* = 4
Mo *K*α radiation
μ = 0.09 mm^−1^
*T* = 93 K
0.20 × 0.07 × 0.03 mm

### Data collection  

Rigaku Saturn724+ diffractometer
Absorption correction: numerical (*NUMABS*; Rigaku, 1999 ▸) *T*_min_ = 0.986, *T*_max_ = 0.997
6174 measured reflections
1795 independent reflections
1457 reflections with *F* ^2^ > 2.0σ(*F* ^2^)
*R*_int_ = 0.046

### Refinement  

*R*[*F* ^2^ > 2σ(*F* ^2^)] = 0.050
*wR*(*F* ^2^) = 0.120
*S* = 1.08
1795 reflections
113 parameters
H atoms treated by a mixture of independent and constrained refinement
Δρ_max_ = 0.22 e Å^−3^
Δρ_min_ = −0.19 e Å^−3^

### 

Data collection: *CrystalClear* (Rigaku, 2008 ▸); cell refinement: *CrystalClear*; data reduction: *CrystalClear*; program(s) used to solve structure: *SHELXD2013* (Sheldrick, 2008 ▸); program(s) used to refine structure: *SHELXL2013* (Sheldrick, 2008 ▸); molecular graphics: *ORTEP-3 for Windows* (Farrugia, 2012 ▸); software used to prepare material for publication: *CrystalStructure* (Rigaku, 2014 ▸).

## Supplementary Material

Crystal structure: contains datablock(s) global, I. DOI: 10.1107/S2056989014028035/hb7326sup1.cif

Structure factors: contains datablock(s) I. DOI: 10.1107/S2056989014028035/hb7326Isup2.hkl

Supporting information file. DOI: 10.1107/S2056989014028035/hb7326Isup3.cml

**Fig. 1.**
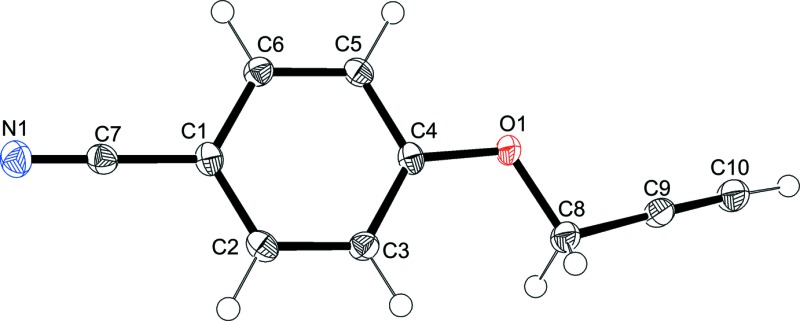
The molecular structure of the title compound with displacement ellipsoids drawn at the 50% probability level and H atoms are shown as small spheres.

. DOI: 10.1107/S2056989014028035/hb7326fig1.tif
The mol­ecular structure of the title compound with displacement ellipsoids drawn at the 50% probability level and H atoms are shown as small spheres.

**Fig. 2.**
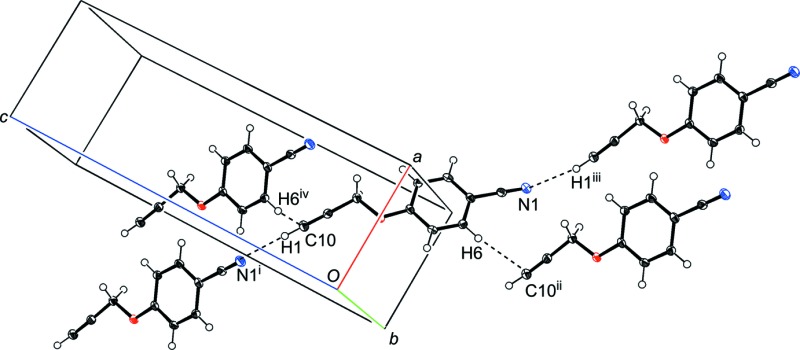
Part of the crystal structure showing the rolling sheet structure formed by the C–H···N and C–H···π hydrogen bonds [Symmetry codes: (i) x - 3/2, -y + 3/2, z + 1/2; (ii) x + 1/2, -y + 3/2, z - 1/2; (iii) x + 3/2, -y + 3/2, z - 1/2; (iv) x - 1/2, -y + 3/2, z + 1/2].

**Fig. 3.**
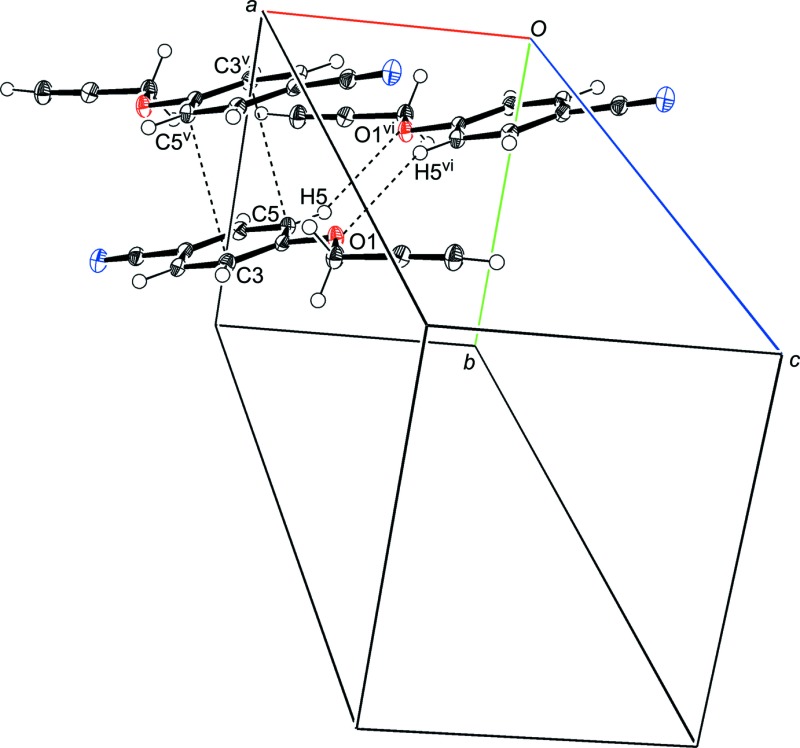
Part of the crystal structure showing the intersheet π···π stacking interactions and the weak C–H···O hydrogen bonds [Symmetry codes: (v) -x + 2, -y + 1, -z; (vi) -x + 1, -y + 1, -z].

Click here for additional data file.x y z x y z x y z x y z . DOI: 10.1107/S2056989014028035/hb7326fig2.tif
Part of the crystal structure showing the rolling sheet structure formed by the C–H⋯N and C–H⋯π hydrogen bonds [Symmetry codes: (i) *x* − 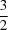, −*y* + 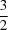, *z* + 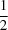; (ii) *x* + 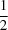, −*y* + 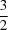, *z* − 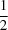; (iii) *x* + 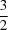, −*y* + 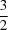, *z* − 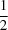; (iv) *x* − 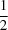, −*y* + 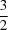, *z* + 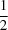].

x y z x y z . DOI: 10.1107/S2056989014028035/hb7326fig3.tif
Part of the crystal structure showing the inter­sheet π⋯π stacking inter­actions and the weak C–H⋯O hydrogen bonds [Symmetry codes: (v) −*x* + 2, −*y* + 1, −*z*; (vi) −*x* + 1, −*y* + 1, −*z*].

CCDC reference: 1041123

Additional supporting information:  crystallographic information; 3D view; checkCIF report

## Figures and Tables

**Table 1:** Hydrogen-bond geometry (, )

*D*H*A*	*D*H	H*A*	*D* *A*	*D*H*A*
C10H1N1^i^	0.94(2)	2.41(2)	3.300(3)	158.18(11)
C6H6C10^ii^	0.95	2.79	3.616(3)	145

Symmetry codes: (i) 
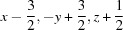
; (ii) 
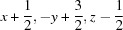
.

## supplementary crystallographic information

### S1. Comment

The title compound, C_10_H_7_N_1_O_1_, is an aryl propargyl ether derivative which attracts interest from viewpoints of Claisen rearrangement (Kenny *et al.* 2006; Wang *et al.* 2012) or cleavage of O–CH_2_ bond by boron reagents (Yao *et al.* 2009). In these reactions, a direction of the lone pair of the oxygen has large influence upon reactivity.

The molecule has an almost planar structure (atoms C1—C10/N1/O1 are essentailly co-planar with r.m.s. deviation = 0.0862 Å), indicating an effective conjugation of the cyano group, the C1—C6 benzene ring and the lone pair of the O1 (Fig. 1). This is presumably because push-pull effect between an electron donating alkyloxy group and an electron withdrawing cyano group (Zhu *et al.* 2006; Marsh 2009; Ranjith *et al.* 2010; Ao *et al.* 2011; Al-Mehana *et al.* 2011; Belay *et al.* 2012; Doi & Okuno 2013).

In the crystal, C10–H1···N1^i^ hydrogen bonds [Symmetry code: (i) *x* - 3/2, -*y* + 3/2, *z* + 1/2] connect the molecules to make a one-dimensional wavy chain. Intermolecular C6–H6···C10^ii^ interaction [Symmetry code: (ii) *x* + 1/2, -*y* + 3/2, *z* - 1/2], whose distance is 2.794 (1) Å, binds the chains to form a rolling sheet structure as shown in Fig. 2.

Fig. 3 shows π···π stacking interactions between the sheets, where the centroid to centroid distance is 3.593 (2) Å and the C3···C5^v^ is 3.387 (3) Å [Symmetry code: (v) -*x* + 2, -*y* + 1, -*z*]. The molecules also form weak intersheet C5–H5···O1^vi^ bonds whose distance is 2.690 (1) Å [Symmetry code: (vi) -*x* + 1, -*y* + 1, -*z*]. In this crystal, the intermolecular hydrogen bonds, the C–H···π interactions and the π···π stacking interactions are found to make a three-dimensional molecular network.

### S2. Experimental

The title compound is commercially available. Colourless platelets of sufficient quality for diffraction measurements were prepared by sublimation at room temperature.

### S3. Refinement

The C-bound H atoms except for C*_sp_*—H were placed at ideal positions and were refined as riding on their parent C atoms. *U*_iso_(H) values of the H atoms were set at 1.2*U*_eq_(parent atom). The C*_sp_*-bound H atom was obtained from a difference Fourier map and was refined isotropically without any restrictions.

### Crystal data

**Table:**

C_10_H_7_NO	*F*(000) = 328.00
*M**_r_* = 157.17	*D*_x_ = 1.335 Mg m^−^^3^
Monoclinic, *P*2_1_/*n*	Mo *K*α radiation, λ = 0.71075 Å
*a* = 6.033 (4) Å	Cell parameters from 2559 reflections
*b* = 7.393 (5) Å	θ = 2.3–31.1°
*c* = 17.527 (11) Å	µ = 0.09 mm^−^^1^
β = 90.836 (11)°	*T* = 93 K
*V* = 781.7 (9) Å^3^	Platelet, colorless
*Z* = 4	0.20 × 0.07 × 0.03 mm

### Data collection

**Table:**

Rigaku Saturn724+ diffractometer	1457 reflections with *F*^2^ > 2σ(*F*^2^)
Detector resolution: 28.445 pixels mm^-1^	*R*_int_ = 0.046
ω scans	θ_max_ = 27.5°, θ_min_ = 2.3°
Absorption correction: numerical (*NUMABS*; Rigaku, 1999)	*h* = −7→7
*T*_min_ = 0.986, *T*_max_ = 0.997	*k* = −9→9
6174 measured reflections	*l* = −21→22
1795 independent reflections	

### Refinement

**Table:**

Refinement on *F*^2^	Secondary atom site location: difference Fourier map
*R*[*F*^2^ > 2σ(*F*^2^)] = 0.050	Hydrogen site location: inferred from neighbouring sites
*wR*(*F*^2^) = 0.120	H atoms treated by a mixture of independent and constrained refinement
*S* = 1.08	*w* = 1/[σ^2^(*F*_o_^2^) + (0.0584*P*)^2^ + 0.1471*P*] where *P* = (*F*_o_^2^ + 2*F*_c_^2^)/3
1795 reflections	(Δ/σ)_max_ < 0.001
113 parameters	Δρ_max_ = 0.22 e Å^−^^3^
0 restraints	Δρ_min_ = −0.19 e Å^−^^3^
Primary atom site location: structure-invariant direct methods	

### Special details

**Table:**

Geometry. ENTER SPECIAL DETAILS OF THE MOLECULAR GEOMETRY
Refinement. Refinement was performed using all reflections. The weighted *R*-factor (*wR*) and goodness of fit (*S*) are based on *F*^2^. *R*-factor (gt) are based on *F*. The threshold expression of *F*^2^ > 2.0 σ(*F*^2^) is used only for calculating *R*-factor (gt).

### Fractional atomic coordinates and isotropic or equivalent isotropic displacement parameters (Å^2^)

**Table:**

	*x*	*y*	*z*	*U*_iso_*/*U*_eq_	
O1	0.66646 (16)	0.60053 (14)	0.09599 (6)	0.0181 (3)	
N1	1.4021 (2)	0.95596 (18)	−0.14609 (7)	0.0234 (3)	
C1	1.1274 (2)	0.81486 (19)	−0.05087 (8)	0.0166 (3)	
C2	1.1843 (2)	0.79920 (19)	0.02615 (8)	0.0175 (3)	
C3	1.0358 (2)	0.72770 (19)	0.07719 (8)	0.0168 (3)	
C4	0.8276 (2)	0.67109 (18)	0.05072 (8)	0.0152 (3)	
C5	0.7705 (2)	0.68441 (19)	−0.02634 (8)	0.0162 (3)	
C6	0.9187 (2)	0.75633 (19)	−0.07715 (8)	0.0168 (3)	
C7	1.2805 (2)	0.8928 (2)	−0.10375 (8)	0.0176 (3)	
C8	0.7121 (2)	0.5908 (2)	0.17643 (8)	0.0197 (3)	
C9	0.5068 (2)	0.5386 (2)	0.21392 (8)	0.0190 (3)	
C10	0.3416 (3)	0.4999 (2)	0.24560 (9)	0.0235 (4)	
H1	0.211 (3)	0.479 (3)	0.2728 (11)	0.033 (5)*	
H2	1.32622	0.838	0.04368	0.0210*	
H3	1.07475	0.71712	0.12971	0.0201*	
H5	0.62929	0.64389	−0.0439	0.0195*	
H6	0.8798	0.76623	−0.1297	0.0202*	
H8A	0.76355	0.70976	0.19566	0.0236*	
H8B	0.82942	0.50026	0.18705	0.0236*	

### Atomic displacement parameters (Å^2^)

**Table:**

	*U*^11^	*U*^22^	*U*^33^	*U*^12^	*U*^13^	*U*^23^
O1	0.0144 (5)	0.0253 (6)	0.0147 (5)	−0.0035 (4)	0.0030 (4)	0.0018 (4)
N1	0.0173 (6)	0.0298 (7)	0.0233 (7)	0.0007 (6)	0.0032 (5)	0.0022 (6)
C1	0.0143 (7)	0.0156 (7)	0.0199 (8)	0.0023 (5)	0.0042 (6)	−0.0003 (6)
C2	0.0117 (7)	0.0177 (7)	0.0231 (8)	0.0021 (5)	0.0005 (6)	−0.0021 (6)
C3	0.0146 (7)	0.0199 (7)	0.0158 (7)	0.0018 (5)	−0.0005 (6)	0.0003 (6)
C4	0.0131 (6)	0.0141 (7)	0.0186 (7)	0.0011 (5)	0.0046 (5)	−0.0006 (6)
C5	0.0119 (7)	0.0174 (7)	0.0193 (8)	0.0003 (5)	−0.0005 (5)	−0.0007 (6)
C6	0.0160 (7)	0.0189 (7)	0.0155 (7)	0.0015 (6)	0.0004 (6)	−0.0003 (6)
C7	0.0141 (7)	0.0195 (7)	0.0193 (8)	0.0012 (6)	−0.0001 (6)	−0.0011 (6)
C8	0.0155 (7)	0.0276 (8)	0.0161 (8)	−0.0004 (6)	0.0024 (6)	0.0006 (6)
C9	0.0179 (7)	0.0239 (8)	0.0151 (7)	0.0018 (6)	−0.0002 (6)	0.0005 (6)
C10	0.0182 (7)	0.0339 (9)	0.0185 (8)	−0.0014 (7)	0.0027 (6)	0.0000 (7)

### Geometric parameters (Å, º)

**Table:**

O1—C4	1.3673 (18)	C8—C9	1.463 (2)
O1—C8	1.434 (2)	C9—C10	1.183 (2)
N1—C7	1.150 (2)	C2—H2	0.950
C1—C2	1.393 (2)	C3—H3	0.950
C1—C6	1.403 (2)	C5—H5	0.950
C1—C7	1.438 (2)	C6—H6	0.950
C2—C3	1.380 (2)	C8—H8A	0.990
C3—C4	1.397 (2)	C8—H8B	0.990
C4—C5	1.392 (2)	C10—H1	0.94 (2)
C5—C6	1.378 (2)		
			
C4—O1—C8	117.49 (11)	C1—C2—H2	119.768
C2—C1—C6	119.99 (13)	C3—C2—H2	119.770
C2—C1—C7	120.43 (13)	C2—C3—H3	120.380
C6—C1—C7	119.58 (13)	C4—C3—H3	120.387
C1—C2—C3	120.46 (13)	C4—C5—H5	119.993
C2—C3—C4	119.23 (13)	C6—C5—H5	119.976
O1—C4—C3	124.41 (13)	C1—C6—H6	120.174
O1—C4—C5	114.96 (12)	C5—C6—H6	120.181
C3—C4—C5	120.63 (13)	O1—C8—H8A	110.181
C4—C5—C6	120.03 (13)	O1—C8—H8B	110.187
C1—C6—C5	119.65 (13)	C9—C8—H8A	110.179
N1—C7—C1	179.63 (15)	C9—C8—H8B	110.175
O1—C8—C9	107.64 (12)	H8A—C8—H8B	108.478
C8—C9—C10	178.27 (16)	C9—C10—H1	175.1 (12)
			
C4—O1—C8—C9	171.29 (10)	C1—C2—C3—C4	0.0 (2)
C8—O1—C4—C3	2.52 (18)	C2—C3—C4—O1	−179.13 (12)
C8—O1—C4—C5	−177.34 (10)	C2—C3—C4—C5	0.7 (2)
C2—C1—C6—C5	0.4 (2)	O1—C4—C5—C6	178.94 (10)
C6—C1—C2—C3	−0.6 (2)	C3—C4—C5—C6	−0.9 (2)
C7—C1—C2—C3	178.70 (12)	C4—C5—C6—C1	0.4 (2)
C7—C1—C6—C5	−178.91 (11)		

### Hydrogen-bond geometry (Å, º)

**Table:**

*D*—H···*A*	*D*—H	H···*A*	*D*···*A*	*D*—H···*A*
C10—H1···N1^i^	0.94 (2)	2.41 (2)	3.300 (3)	158.18 (11)
C6—H6···C10^ii^	0.95	2.79	3.616 (3)	145

Symmetry codes: (i) *x*−3/2, −*y*+3/2, *z*+1/2; (ii) *x*+1/2, −*y*+3/2, *z*−1/2.
