# Supplementary figures and images for: Crystal structure of 4-(prop-2-yn-1-yl­oxy)benzo­nitrile
Source: Acta Crystallogr E Crystallogr Commun. 2015 Jan 10;71(Pt 2):o97–8. doi: 10.1107/S2056989014028035 (PMC4384594; doi:10.1107/S2056989014028035)

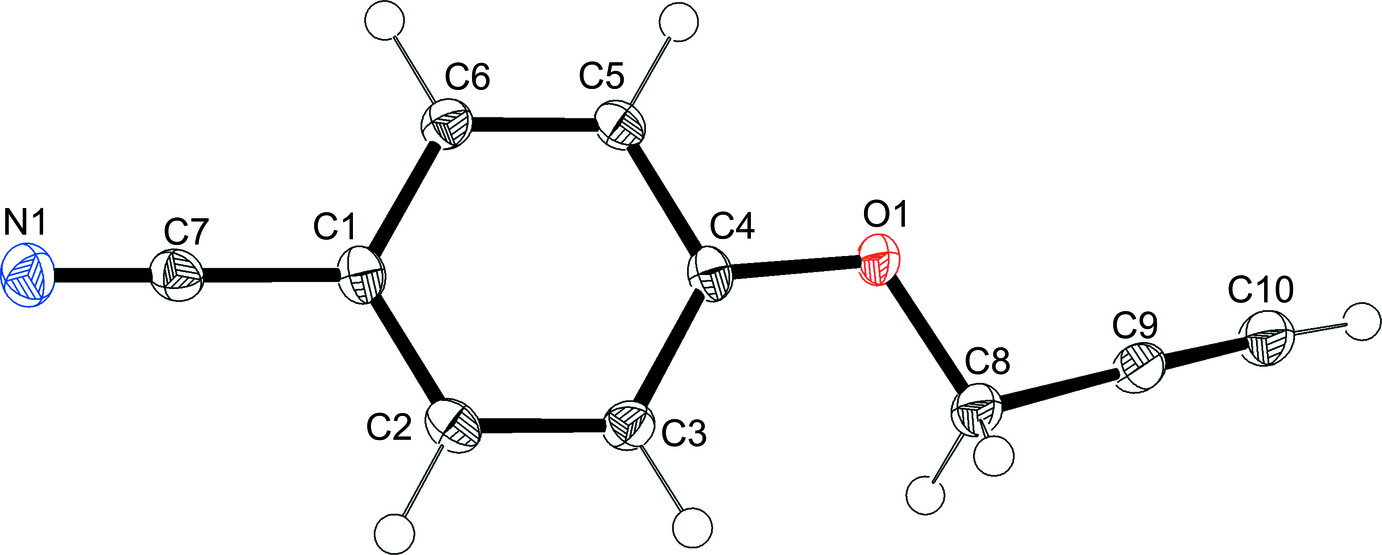

Supplement: Supplementary file 4 [file e-71-00o97-fig1.tif]

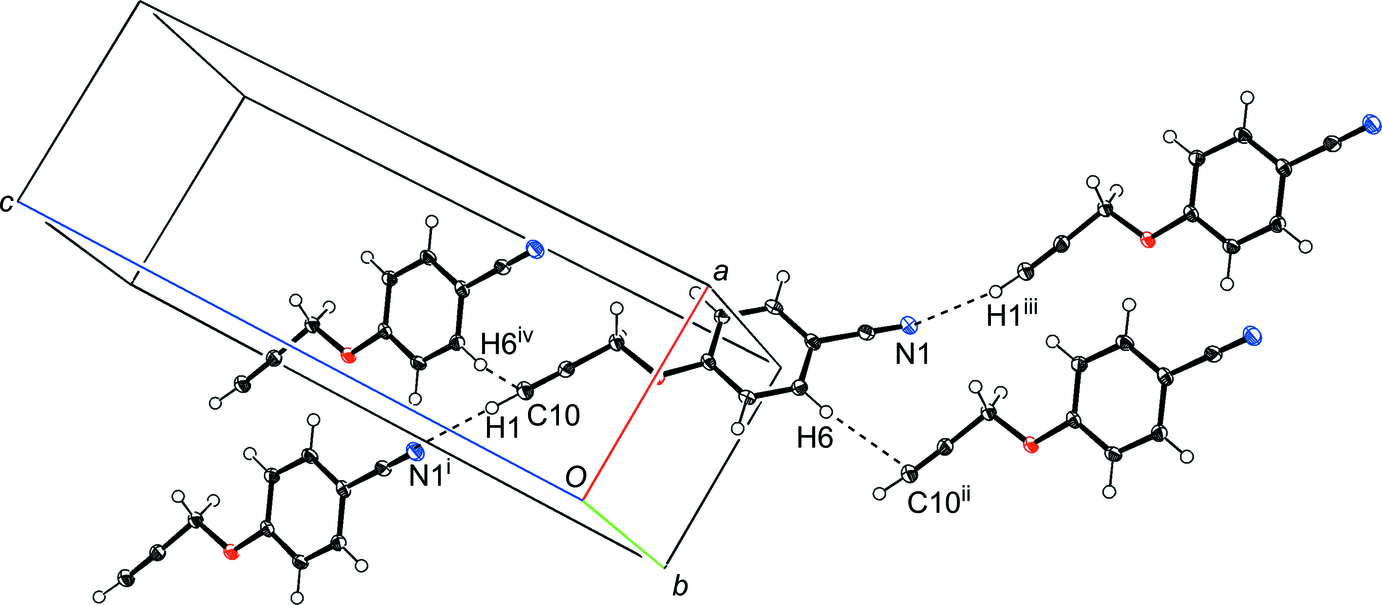

Supplement: Supplementary file 5 [file e-71-00o97-fig2.tif]

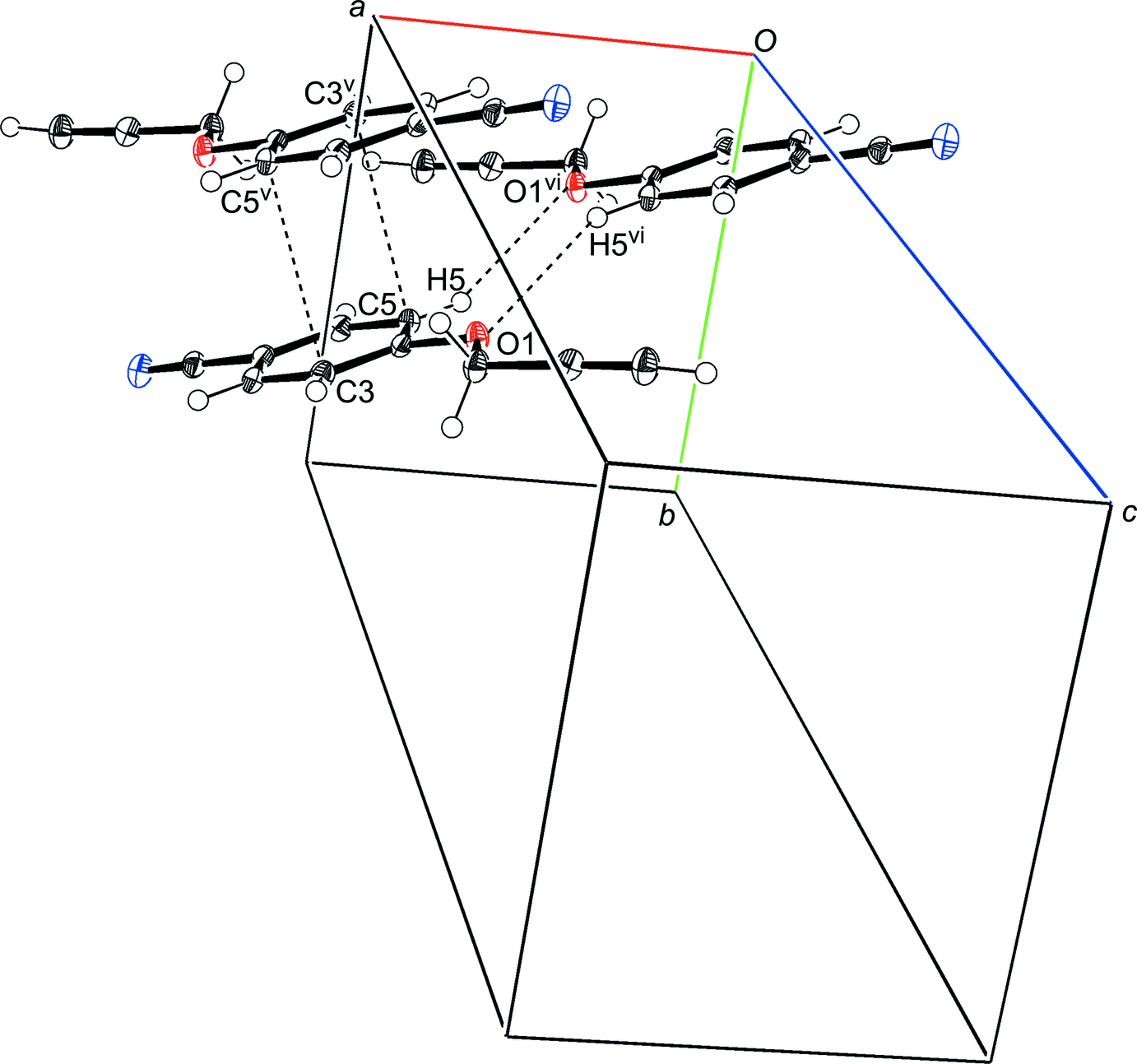

Supplement: Supplementary file 6 [file e-71-00o97-fig3.tif]
